# Expanded Nanofibrous Polymeric Mats Incorporating Tetracycline-Loaded Silica Mesoporous Nanoparticles for Antimicrobial Applications

**DOI:** 10.3390/pharmaceutics17101335

**Published:** 2025-10-15

**Authors:** Federico Fookes, Silvestre Bongiovanni Abel, Josefa F. Martucci, Diana Estenoz, Gustavo A. Abraham, Carlos A. Busatto

**Affiliations:** 1 Group of Polymers and Polymerization Reactors, INTEC (National University of Litoral-CONICET), Güemes 3450, Santa Fe 3000, Argentina; 2 Health Sciences College, UCSF (Santa Fe Catholic University), Echagüe 7151, Santa Fe 3000, Argentina; 3 Biomedical Polymers Division, INTEMA (National University of Mar del Plata-CONICET), Av. Colón 10850, Mar del Plata 7600, Argentina; gabraham@fi.mdp.edu.ar; 4 Department of Chemical and Food Engineering, Faculty of Engineering, National University of Mar del Plata, Av. Juan B. Justo 4302, Mar del Plata 7600, Argentina; 5 Sustainable Materials Division, INTEMA (National University of Mar del Plata-CONICET), Av. Colón 10850, Mar del Plata 7600, Argentina

**Keywords:** expanded electrospun mats, mesoporous silica nanoparticles, tetracycline, drug release, antimicrobial activity

## Abstract

**Background/Objectives:** In this work, expanded electrospun poly(vinyl alcohol) (PVA) nanofiber mats incorporating tetracycline-loaded mesoporous silica nanoparticles (MSNs) were fabricated for antimicrobial wound dressing applications. **Methods:** MSNs with high surface area were synthesized and efficiently loaded with tetracycline, achieving sustained drug release. These nanoparticles were then embedded into both conventional (2D) and gas-expanded (3D) electrospun PVA mats. **Results:** The gas-foaming process significantly enhanced the mat’s thickness, promoting improved nanoparticle loading and diffusion properties. Physicochemical characterization confirmed the structural integrity, thermal stability, and successful drug incorporation within the hybrid scaffolds. Antimicrobial tests against *Escherichia coli* and *Staphylococcus aureus* demonstrated excellent bactericidal effects, with superior inhibition observed in 3D mats due to their higher drug loading capacity and faster drug release related to the expanded structure. **Conclusions:** These results highlight the potential of combining electrospinning, gas expansion, and nanocarriers to engineer advanced, drug-loaded fibrous scaffolds for wound healing.

## 1. Introduction

The skin is the largest organ of the human body, serving as the first barrier against physical, chemical, and biological damage [1]. Treating skin injuries, including wounds, ulcers, and burns, requires temporary barriers that prevent microbial infections, allow gas exchange, absorb excess exudate, maintain a moist environment to promote epithelial growth, and minimize both scarring and patient discomfort [2,3]. Traditional wound dressings, such as gauze and tulle, provide only passive protection and can adhere to the wound, potentially causing further epithelial damage when removed [4]. In this regard, active wound dressings that can promote healing have been developed based on synthetic polymers such as poly(lactic-co-glycolic) acid [5], polyurethane [6], poly-ε-caprolactone [7], poly ethylene glycol [8], and polyvinyl alcohol, as well as natural biomaterials including alginate [9], chitin [10], chitosan [11], collagen [12], gelatin [13], hyaluronic acid [14], and silk fibroin [15]. The scaffolds for wound healing have been used in various forms, such as fibrous networks, sponges, foams, and hydrogels, among others [16]. However, many of these scaffolds fail to replicate the structural complexity of the skin’s extracellular matrix (ECM) [17], which can affect the wound healing efficiency.

In recent years, electrospun polymeric nanofibers have emerged as promising materials for wound healing due to their ability to mimic the extracellular environment of tissues, leading to enhanced cellular adhesion and proliferation [18,19]. In addition, their nanometer-scale fiber diameters, high surface-to-volume ratios, and interconnected porosity are particularly advantageous for tissue engineering applications [20]. However, their two-dimensional structure, dense fiber packing, limited thickness, and small pore sizes restrict their potential for wound healing [19]. In this regard, the complex three-dimensional (3D) structure and functionality of the ECM remain significant challenges for synthetic tissue engineering. Among the strategies developed to address these limitations, gas expansion technology has shown promise in generating porous 3D matrices [21,22]. This technique involves the introduction of an expansion agent into a polymeric solution to trap gas bubbles, forming pores within the matrix. Combining electrospinning with gas expansion techniques offers a means to increase pore size and interconnectivity [23]. The 3D scaffolds not only provide improved water retention, but also offer an additional dimension that promotes cell-to-cell interactions, supports cell migration, and enhances the diffusion of nutrients and metabolites [22]. The design of next-generation wound dressings must integrate key properties such as ECM-mimicking morphology, mechanical integrity, exudate absorption, and antibacterial activity [24].

Polyvinyl alcohol (PVA) is a hydrophilic polymer with remarkable properties for preparing nanofibrous wound dressings, including biodegradability, biocompatibility, non-toxicity, chemical resistance, moisture absorbency, and excellent thermal stability [25,26]. In addition, several studies have demonstrated the potential of active compounds in nanofibrous PVA scaffolds for wound healing. In this direction, Amer et al. explored the use of total ethanol extract (TEE) and polysaccharides (Poly) from *Lepidium sativum* L. seeds, loaded into PVA nanofibers (NFs), for wound healing applications. The results showed that TEE exhibited stronger antioxidant activity and better wound healing efficacy than Poly, suggesting that TEE-loaded NFs are more effective for use in wound dressings [27]. Nemati et al. developed PVA nanofibers loaded with zinc oxide nanoparticles and curcumin, showing significant antioxidant and antibacterial effects, as well as controlled curcumin release over 7 days. In vivo testing on rats revealed that the PVA dressing promoted wound contraction [25]. Mahmoudi et al. developed a biocompatible fibrous scaffold composed of PVA, bioactive glass, silver nanoparticles, and curcumin using electrospinning, demonstrating favorable mechanical properties, high fibroblast viability, and excellent antibacterial activity [28]. While controlled-release formulations have advanced significantly in recent decades, challenges remain, particularly regarding the efficient release of hydrophilic drugs. Developing a system capable of delivering antibiotics locally and in a controlled manner is therefore of great interest for improving wound treatment.

Mesoporous silica nanoparticles (MSNs) have gained increasing attention as promising platforms in biomedical applications due to their unique properties, such as tunable particle and pore sizes, biocompatibility, high surface area and porosity, versatile surface functionalization, and high drug-loading capacity [29,30]. Drug-loaded MSNs can be incorporated into PVA nanofibers to create multifunctional membranes with sustained drug release. In this direction, Rathinavel et al. investigated the incorporation of SBA-15 mesoporous silica into PVA nanofiber for the delivery of curcumin. The in vitro drug release study demonstrated a sustained release of curcumin for 84 h from the SBA-15-incorporated PVA nanofiber. In addition, it was demonstrated that the material exhibited positive effects on both Gram-positive and Gram-negative bacteria, as well as effective wound-healing activity [26]. In this context, the development of composite wound dressing materials presents a promising strategy for the sustained release of hydrophilic antibiotics such as tetracycline hydrochloride, a broad-spectrum antibiotic with low toxicity that can promote fibroblast adhesion [31]. Additionally, combining electrospinning and gas expansion techniques offers the potential to modify the material structure, adding a new dimension to traditionally flat electrospun materials for wound healing. Thus, this study integrates MSNs loaded with a hydrophilic antibiotic (tetracycline) into gas-expanded three-dimensional electrospun PVA mats. While MSNs and electrospun PVA fibers have individually been explored for wound healing, their combination with a gas-foaming expansion step to generate highly porous 3D antimicrobial scaffolds represents a novel strategy. This approach allows for enhanced drug loading, controlled release, and improved antimicrobial efficacy, thereby addressing current limitations in the design of advanced wound dressings.

This work aims to investigate a novel wound dressing material based on expanded nanofibrous membranes incorporating MSNs for the controlled release of tetracycline, a broad-spectrum antibiotic. The fibrous materials were prepared by electrospinning of PVA, followed by thermal crosslinking and gas expansion to create mechanically stable and porous materials with a 3D structure. Additionally, MSNs were synthesized and used for encapsulating and controlling the release of tetracycline. The tetracycline-loaded MSNs were incorporated into 2D and 3D nanofibrous dressings by infiltration to create multifunctional materials for wound healing. The antimicrobial properties of the wound dressings were tested against both Gram-positive and Gram-negative bacteria, showing excellent antimicrobial activity for both expanded and non-expanded nanofibrous materials.

## 2. Materials and Methods

### 2.1. Materials

Poly(vinyl alcohol) (PVA, Mw = 150,000 g mol^−1^), cetyl trimethyl ammonium bromide (CTAB), and sodium borohydride (NaBH_4_) were purchased from Sigma-Aldrich (St. Louis, MO, USA). Tetraethoxysilane (TEOS) was acquired from Fluka (Neu-Ulm, Germany). Sodium hydroxide, ammonia solution, ethanol, methanol, acetonitrile, and hexane were purchased from Cicarelli (Santa Fe, Argentina). Citric acid was purchased from Anedra (Buenos Aires, Argentina). All chemicals were analytical grade and used as received without further modification. Ultrapure water obtained from a Millipore system (18 MΩ cm of resistivity) was used to prepare all solutions.

### 2.2. Methods

#### 2.2.1. Synthesis of MSN

MCM-41 type mesoporous silica nanoparticles were synthesized according to the procedure described by Williams et al. [32]. Briefly, 384 mL of water was mixed with 2.8 mL of 2 mol L^−1^ sodium hydroxide solution and 800 mg of CTAB. Subsequently, 4 mL of TEOS was added dropwise to the solution, which was then stirred at 80 °C for 2 h. Once the mixture cooled to room temperature, the resulting particles were filtered, washed with water until reaching neutral pH, and dried at 60 °C. To eliminate the residual surfactant, the particles were calcined at 550 °C for 4 h.

#### 2.2.2. PVA Electrospinning

Electrohydrodynamic experiments were carried out using an electrospinning device (Yflow model 2.2.D-350, Málaga, Spain) in single-nozzle mode. The PVA solution was prepared by dissolving the polymer in distilled water at a concentration of 20 wt% under stirring at 300 rpm and heating at 90 °C to ensure complete dissolution. After dissolution, citric acid (5 wt% relative to PVA) was added, and the solution was stirred for 24 h at room temperature. The ground flat collector was fixed at 15 cm from the needle tip, and the polymeric solution was pumped at a flow rate of 0.3 mL h^−1^. The applied voltage was set at 18.5 kV, and environmental parameters were constant (temperature 25 ± 1 °C and relative humidity 35 ± 3%). The process was carried out for 6 h, and then the electrospun sample was dried under vacuum for 48 h. In the next step, thermal crosslinking of mats was performed in an oven (model 52411-30, Cole Parmer, Vernon Hills, Illinois, USA), maintaining the electrospun mats at a controlled temperature (185 ± 5 °C) for 10 min.

#### 2.2.3. Characterization

##### Dynamic Light Scattering and Surface Z-Potential

The size distribution of the nanoparticles was determined by dynamic light scattering (DLS) using a BI–200SM instrument (Brookhaven, Nashua, NH, USA). Measurements were taken at a detection angle of 90° and a temperature of 30 °C. Zeta potential measurements were conducted at neutral pH using a Zetasizer Nano-series Malvern (ZS90) instrument (Malvern Panalytical, Malvern, Worcestershire, UK). Suspensions of MSN at a concentration of 1 g/L were prepared in deionized water and subjected to 10 min of sonication.

##### Electronic Microscopy

Scanning electron microscopic observation of MSNs and PVA electrospun fibers was conducted in a FE-SEM ZEISS Crossbeam 350 microscope (ZEISS, Oberkochen, Germany) at several magnifications after chromium sputtering. The average electrospun fiber diameter was determined from 100 measurements for each image using ImagePro Plus 6.0 software (Media Cybernetics Inc, Silver Spring, MD, USA). The mesoporous structure of MSN was examined by transmission electron microscopy (TEM). Particles were placed on a carbon-coated copper grid, air-dried and observed using a JEOL-2100 Plus electron microscope (JEOL, Tokyo, Japan) at an accelerating voltage of 200 kV.

##### Textural Properties

Textural characteristics were evaluated through nitrogen adsorption analysis using a Micromeritics ASAP 2020 Plus sorptometer (Micromeritics Instrument Corporation, Norcross, GA, USA). Prior to analysis, the samples were degassed overnight at 100 °C. The surface area and average pore diameter were determined using the Brunauer–Emmett–Teller (BET) and Barrett–Joyner–Halenda (BJH) methods, respectively. Total pore volume was estimated at a relative pressure (*p/po*) of approximately 0.99. Micropore volume and the mesopore-specific surface area were obtained using the t-plot method within the thickness range of 3.5 to 5.0 Å.

##### FTIR Spectroscopy

FTIR spectra of tetracycline, MSN, and tetracycline-loaded MSN were recorded using a Fourier transform spectrophotometer (Shimadzu 8201, Kyoto, Japan) within the frequency range of 400 to 4000 cm^−1^, with a spectral resolution of 4 cm^−1^ and 60 scans per sample. Around 3 mg of each sample was blended with 100 mg of dry potassium bromide (KBr), finely ground into a uniform powder, and then pressed into a disc for analysis.

##### Thermal Analysis

The thermal behavior of tetracycline, MSN, and tetracycline-loaded MSN was analyzed using a Q500 TA instrument (TA Instruments, New Castle, DE, USA). The measurements were conducted under a nitrogen atmosphere with a flow rate of 10 mL/min. Approximately 5 mg of each sample was heated from 25 °C to 800 °C at a constant rate of 10 °C/min.

##### Mechanical Analysis

Mechanical tensile tests were carried out using a Labthink C610H auto tensile tester (Labthink Instruments Co., Ltd., Jinan, China) equipped with a 50 N load cell and at a crosshead speed of 10 mm min^−1^, following the procedure described in the ASTM D638-14. Electrospun mats were cut into rectangular strips (0.5 cm × 3.0 cm) and conditioned at 65 ± 2% RH and 23 ± 2 °C before testing. Results are the average of 7 replicates. Mechanical properties (i.e., Young’s modulus (E), tensile strength, and elongation at break were determined on stress versus strain diagrams.

##### Gas-Foaming Procedure

3D PVA electrospun mats were obtained following the procedure recently published by Bongiovanni Abel et al. [33]. Briefly, the samples were cut using a biopsy punch, obtaining discs of 5 mm in diameter. Then, the samples were immersed in 1 mol L^−1^ NaBH_4_ aqueous solution at 25 ± 1 °C for 6 h. After that, the expanded electrospun mat pieces were carefully removed from the solution and rinsed with deionized water. The evolution of the mats’ thickness was measured using an induction sensor digital electronic caliper (Mitutoyo, Kawasaki, Japan). Finally, samples were freeze-dried for 48 h.

##### Tetracycline Loading into MSN

An amount of 500 mg of MSN was dispersed in 25 mL of a saturated tetracycline solution (4.5 mg/mL) in water. The suspension was stirred for 24 h, after which it was centrifuged for 15 min at 10,000 rpm. The supernatant was removed, and the resulting tetracycline-loaded particles were lyophilized and stored until further characterization. To determine the encapsulation efficiency, 5 mg of MSN were resuspended in 30 mL of water, and the suspension was sonicated for 5 min and stirred for 48 h. Tetracycline concentration in the supernatant was subsequently measured using a UV–vis spectrometer (Lambda 25, Perkin Elmer, Shelton, CT, USA) at a wavelength of 275 nm.

##### Tetracycline Release Assay

Approximately 20 mg of tetracycline-loaded MSN or electrospun mats were dispersed in 100 mL of PBS buffer, and the vials were incubated at 37 °C. To enable sampling, the nanoparticles were enclosed in a dialysis membrane (Cellu·Sep T1, DONGIL BIOTECH, Seoul, Republic of Korea, nominal molecular weight cut-off: 3500 Da). At predetermined time intervals, 5 mL of the release medium was collected and replaced with an equal volume of fresh medium. Tetracycline concentrations were determined using a UV–vis spectrometer (Lambda 25, Perkin Elmer, USA) at a wavelength of 275 nm. A calibration curve was generated by preparing tetracycline aqueous solutions over the concentration range of 0–25 mg/L.

##### MSN Infiltration into PVA Mats

Tetracycline-loaded MSN were dispersed in acetone at a concentration of 10 wt%. After that, the 2D or 3D PVA mats (ca. 1 mg) were immersed in the particle dispersion, and the mixture was subjected to sonication for 15 min. The solvent was evaporated in an oven at 40 °C until at a constant weight, and the materials were lyophilized. A visible color change of the original white PVA mats to that of the particle dispersions was observed, indicating successful incorporation of the particles. To determine the encapsulation efficiency of tetracycline in the 2D and 3D PVA mats, approximately 5 mg of tetracycline-loaded electrospun mats were incubated in 10 mL of water, and the suspension was sonicated for 5 min and stirred for 48 h. The concentration of tetracycline in the supernatant was subsequently measured using a UV–vis spectrometer (Lambda 25, Perkin Elmer, USA) at a wavelength of 275 nm.

##### Antimicrobial Studies

The antimicrobial activity of the electrospun mats against *Escherichia coli* and *Staphylococcus aureus* was evaluated using the agar diffusion method by measuring the diameter of the inhibition halos produced by the mats. Each type of membrane, with a diameter of 5 mm, was placed on the surface of tryptic soy agar in Petri dishes that had been previously inoculated with either *Escherichia coli* ATCC 8739 or *Staphylococcus aureus* ATCC 29213. The assay was performed in triplicate. The inoculum was prepared using the direct colony suspension method in saline solution to achieve a turbidity of 0.5 on the McFarland scale, which corresponds to an approximate concentration of 1.5 × 10^8^ CFU mL^−1^. The inoculum was applied using a swab soaked in the standardized suspension and was evenly distributed across the surface of the solidified culture medium. The 5 mm mats were then placed onto the inoculated agar surfaces. The Petri dishes were subsequently incubated at 37 °C for 24 h. Following incubation, the presence or absence of an inhibition halo around the films was recorded. In cases where an inhibition halo was observed, a sample from the inhibition halo was collected and re-cultured on tryptic soy agar plates, which were then incubated at 37 °C for another 24 h. Post-incubation, the presence or absence of bacterial growth was assessed to determine whether the films exhibited bactericidal or bacteriostatic activity. The absence of growth indicated a bactericidal effect, whereas the presence of growth suggested a bacteriostatic effect.

The minimum inhibitory concentration (MIC) and minimum bactericidal concentration (MBC) of tetracycline released from the electrospun mats were determined against *E. coli* and *S. aureus* using the broth macrodilution method [34]. For each assay, a series of 12 sterile Eppendorf tubes was prepared, each containing 1 mL of tryptic soy broth (TSB). One additional tube containing only TSB served as the negative control. Serial twofold dilutions of tetracycline were performed across the tubes, followed by inoculation with a standardized bacterial suspension. Inocula were prepared by suspending colonies (18–24 h old) in sterile saline (0.85% NaCl) and adjusting turbidity to a 0.5 McFarland standard. Tubes were incubated at 37 °C for 24 h, after which bacterial growth was visually assessed. Tubes without visible turbidity were subcultured on tryptic soy agar plates to confirm bactericidal activity and to determine MIC and MBC values.

#### 2.2.4. Statistical Analysis

Each value represents the mean of three separate experiments, and the error bar denotes the standard deviation. Statistically significant differences were established by one-way ANOVA. Values were statistically significant considering a confidence level of 95% (*p <* 0.05).

## 3. Results

**Figure 1:**
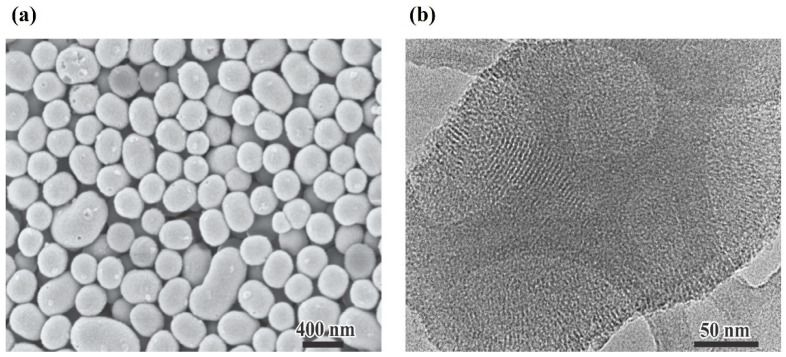
Morphological characterization of MSN by SEM (**a**) and TEM (**b**). Scale bar: (**a**) 400 nm; (**b**) 50 nm.

As a first step, MSN were synthesized and subsequently characterized in terms of their particle size, Z potential, morphology and textural properties. As shown in **Figure 1**, the nanoparticles predominantly exhibit a spherical morphology, although some particles have an ellipsoidal shape. The MSN present an average particle size of 304 ± 35 nm and a Z potential value of −36.20 ± 1.15 mV. The TEM micrographs also revealed a highly ordered mesoporous structure, indicating the successful formation of a uniform internal pore network. Nitrogen adsorption–desorption analysis showed that the nanoparticles displayed Type IV isotherms with a distinct hysteresis loop, which is characteristic of mesoporous materials. The textural properties of MSN are shown in **Table 1**. The results confirm the presence of well-defined mesopores and a high surface area, both of which are desirable features for high-capacity loading and controlled release of tetracycline.

**Figure 2:**
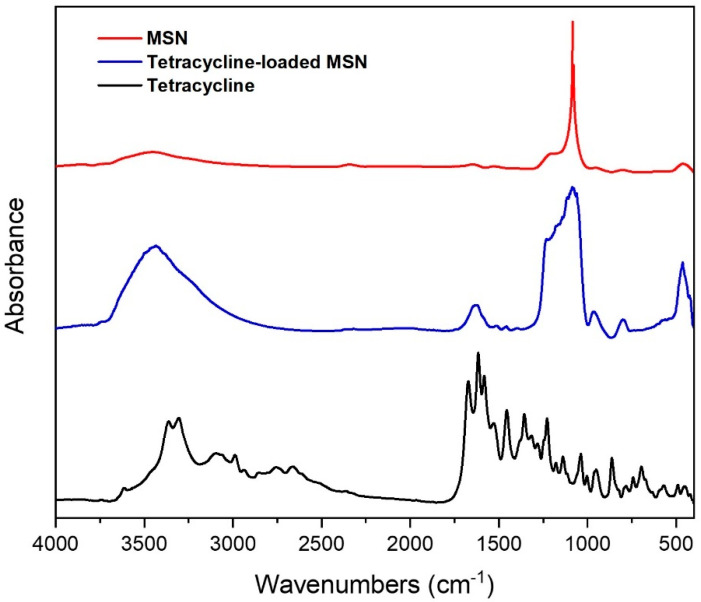
FTIR spectra of MSN, tetracycline, and tetracycline-loaded MSN.

The MSN were employed to study the tetracycline loading. The tetracycline-loaded nanoparticles were characterized by FTIR analysis to determine the characteristic groups of the drug and the delivery system. **Figure 2** presents the FTIR spectra of MSN, tetracycline, and tetracycline-loaded MSN. The FTIR spectrum of MSN exhibits an absorption peak at 800 cm^−1^; and an intense band at 1086 cm^−1^, which correspond to the symmetric and asymmetric stretching vibrations of Si–O–Si, respectively [35]. Additionally, a low-intensity peak attributed to Si–OH bending appears at 962 cm^−1^;. For tetracycline, a broad absorption band is observed in the region of 3200–3500 cm^−1^, which is attributed to N–H and O–H stretching. In addition, intense and sharp peaks appear around 1650–1700 cm^−1^, related to the carbonyl (C=O) stretching vibrations. Peaks in the region of 1400–1600 cm^−1^ are typically assigned to C=C stretching vibrations of the aromatic rings within the tetracycline structure [36]. Absorptions in the range of 1000–1300 cm^−1^ correspond to C–N stretching in amines and C–O stretching in alcohols and ethers. When tetracycline was loaded into the MSN, its characteristic absorption bands were clearly observed in the FTIR spectrum of the tetracycline-loaded MSN, appearing at approximately 3435, 1632, 1230, and 965 cm^−1^. These bands correspond to the O–H/N–H stretching, C=O stretching, C–N and C–O stretching, and N–H bending vibrations, respectively. The presence of these distinct tetracycline-related bands in the FTIR spectrum of the MSN formulation suggests successful drug incorporation. This finding is consistent with the high surface area and pore volume of MSN, which facilitate the adsorption and retention of drug molecules through hydrogen bonding and physical entrapment.

**Figure 3:**
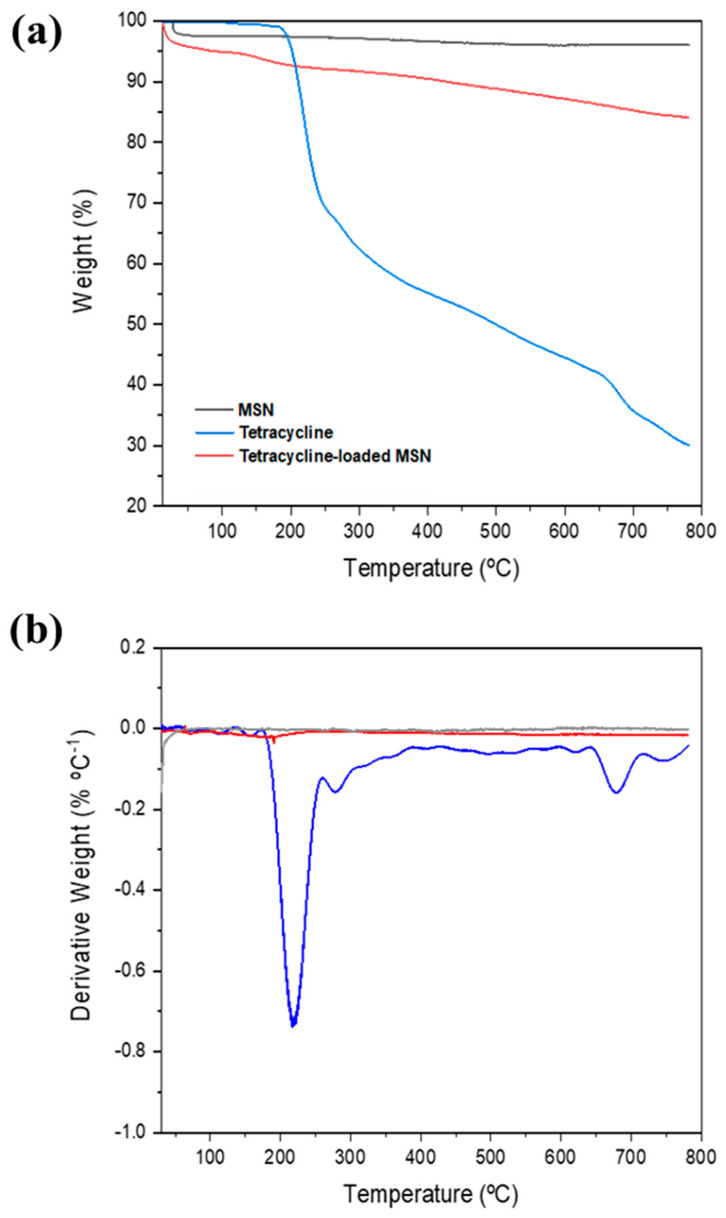
(**a**) Thermogravimetric (TG) profiles of tetracycline, MSN, and tetracycline-loaded MSN. (**b**) Corresponding derivative thermogravimetric (DTG) curves.

Thermogravimetric analysis (TGA) demonstrated excellent thermal stability of the MSN throughout the examined temperature range, which decreased with the drug incorporation in the nanoparticles. The thermogravimetric profiles of MSN, tetracycline, and tetracycline-loaded MSN are illustrated in **Figure 3**. Tetracycline showed a weight loss event starting at approximately 200 °C, corresponding to its thermal degradation. Following this rapid mass loss, a gradual degradation process was observed. At the maximum temperature of 780 °C, tetracycline retained approximately 30% of its original mass. The TGA curve of tetracycline-loaded MSN presents an initial mass loss attributed to the evaporation of residual water and resembles that of tetracycline. However, the overall mass loss was significantly lower, reflecting the minimal thermal decomposition of the silica nanoparticles. From these observations, the tetracycline loading on MSN was estimated to be approximately 9% (*w*/*w*). The high BET surface area and well-ordered mesoporous structure of the synthesized MSNs rationalize the efficient tetracycline loading. Mesoporous silica materials are widely recognized for their tunable pores, large surface areas and high loading capacity, which favor the adsorption and diffusion-controlled release of small antibiotics such as tetracycline [37].

**Figure 4:**
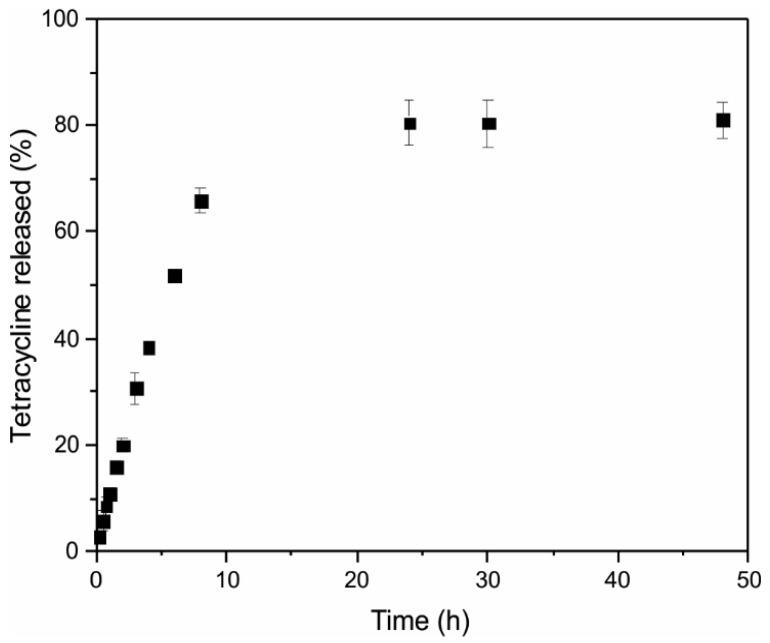
In vitro tetracycline release profile from MSN.

The in vitro release profile of tetracycline from the MSN demonstrated a sustained release behavior over a 24-h period (**Figure 4**). A minimal initial burst release was observed within the first few hours, which is attributable to the desorption of surface-adsorbed tetracycline. This was followed by a gradual and continuous release phase, indicating the effective encapsulation of the drug within the nanoparticles and controlled diffusion through the inorganic matrix. The sustained release pattern suggests that the nanoparticles effectively modulate the release kinetics of tetracycline, potentially minimizing the frequency of administration. The extended-release profile is advantageous for maintaining drug concentrations within the therapeutic window over an extended duration, thereby reducing the risk of resistance development commonly associated with sub-therapeutic levels of antibiotics.

**Figure 5:**
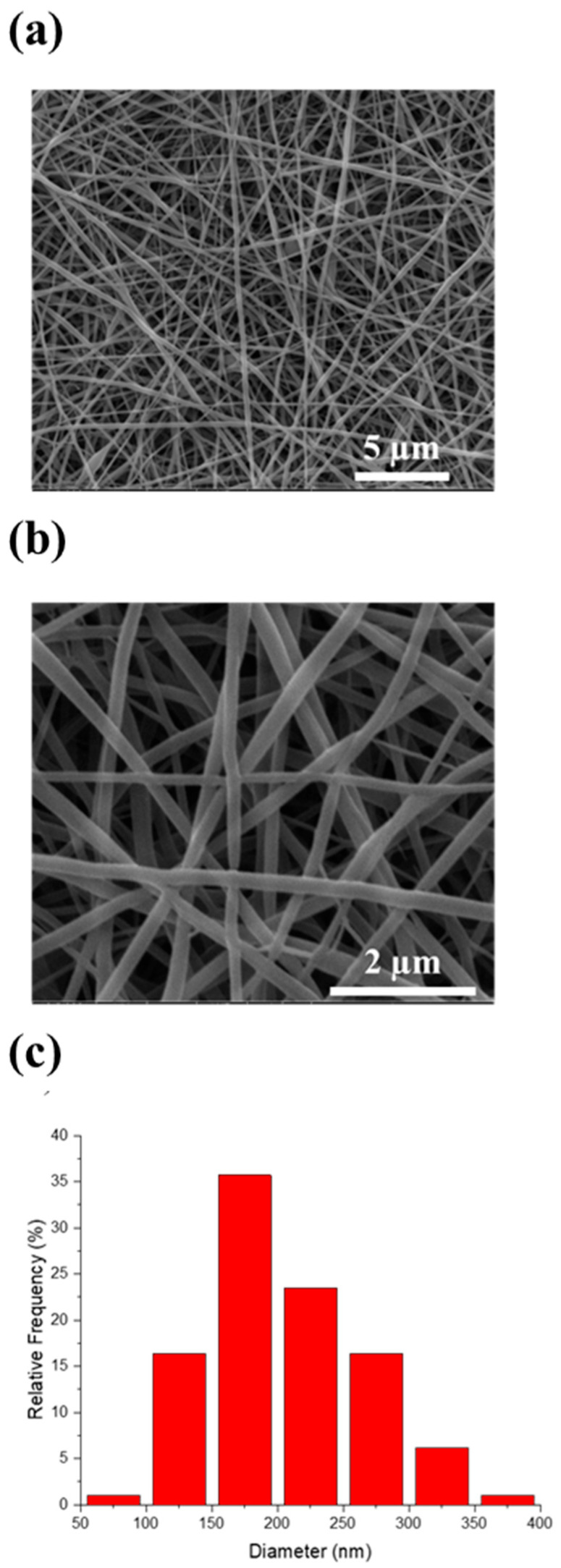
SEM micrographs 2D PVA mats at (**a**) 10000×, and (**b**) 40000×; (**c**) fiber diameter distribution of nanofibers.

The experimental conditions for the preparation of PVA electrospun mats were optimized to obtain homogeneous and reproducible PVA membranes, featuring randomly deposited, defect-free fibers (**Figure 5**). To prevent dissolution of the PVA fibers in aqueous media, a thermal crosslinking process was performed as described in the experimental section. Based on scanning electron microscopy (SEM) micrographs (**Figure 5a**,**b**), the average fiber diameter was determined to be 202 ± 60 nm. The fiber size distribution is presented in **Figure 5c** as a percentage relative frequency after counting fibers.

**Figure 6:**
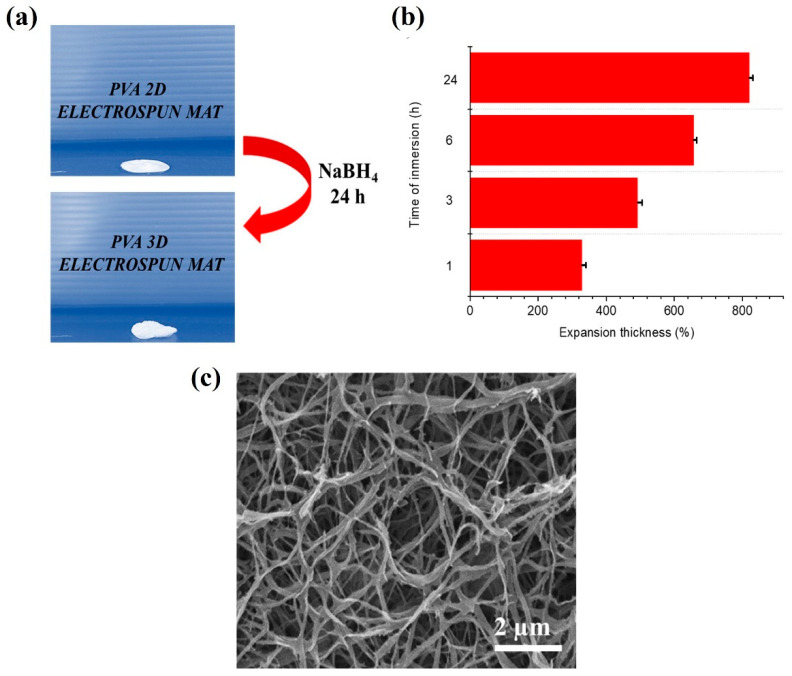
(**a**) Photographs comparing the PVA 2D and 3D electrospun mats (sample size: 5 mm in diameter); (**b**) Increment of PVA thickness mats as a function of immersion time in 1 mol L^−1^ NaBH_4_ solution; (**c**) SEM micrograph of a 3D PVA electrospun mat after gas foaming procedure.

The electrospun mats underwent an expansion treatment involving the incorporation of sodium borohydride, a chemical agent known for its gas-releasing properties upon reaction with water. This process was implemented to enhance both the porosity and the interconnectivity of the fibrous structure [23,38]. As illustrated in **Figure 6a**,**b**, the expanded materials demonstrated a noticeable increase in thickness, which can be attributed to the formation of larger pore sizes and the improved inter-fiber connectivity within the matrix. This was also confirmed by SEM observations (**Figure 6c**). These morphological changes are particularly advantageous in the context of wound healing applications, as they facilitate more effective exudate absorption. An expanded and highly porous structure allows for greater fluid uptake and retention, which is essential for maintaining a moist wound environment, promoting tissue regeneration, and reducing the risk of infection.

**Figure 7:**
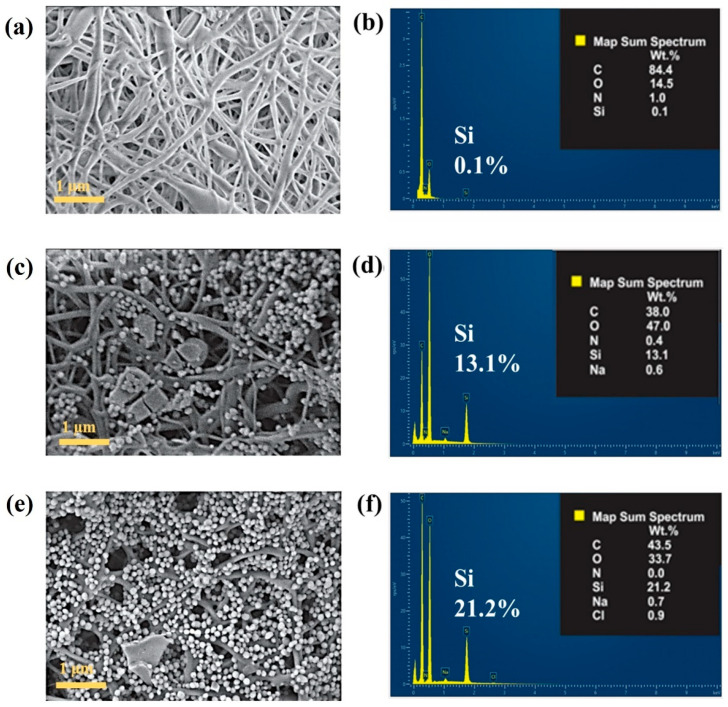
SEM and EDS analyses of electrospun mats incorporating tetracycline-loaded MSN: (**a**,**b**) Non-loaded PVA scaffold; (**c**,**d**) MSN-loaded 2D PVA scaffold; (**e**,**f**) MSN-loaded 3D PVA scaffold.

In a subsequent step, the infiltration of tetracycline-loaded MSN into the PVA mats was investigated. For this purpose, a concentrated dispersion of drug-loaded nanoparticles was prepared and used to incubate both the non-expanded (2D) and expanded (3D) PVA electrospun mats. The successful incorporation of nanoparticles into the fibrous scaffolds was confirmed through SEM and EDS analyses (**Figure 7**), as well as by quantitative assessment of drug encapsulation. SEM micrographs revealed the presence of nanoparticles predominantly located within the interfibrous spaces, which is likely due to their size being compatible with the dimensions of the pores and fiber gaps. Notably, the 3D expanded scaffolds exhibited a visibly higher concentration of embedded nanoparticles compared with their 2D counterparts, a phenomenon attributed to their enhanced porosity and interconnectivity resulting from the expansion treatment. This observation was further substantiated by the drug encapsulation data, which demonstrated tetracycline loadings of 0.85 ± 0.35 wt% for the 2D mats and 1.95 ± 0.43 wt% for the 3D mats. Additionally, EDS analysis of PVA and hybrid scaffolds confirmed the presence of Si atoms from MSN in the hybrid scaffold. The atomic percentages of Si were 0.1, 13.1, and 21.2% for the control PVA scaffold, the drug-loaded 2D PVA scaffold and the drug-loaded 3D PVA scaffold, respectively. EDS measurements also reveal the presence of Cl atoms in the hybrid 3D PVA scaffolds, which are attributed to tetracycline hydrochloride. These findings demonstrate the effectiveness of the expansion process in facilitating a greater incorporation of drug-loaded MSN, likely due to the increased internal surface area and the improved accessibility of the porous network within the 3D scaffold structure.

Mechanical characterization of the mats was performed under uniaxial tensile loading, with representative stress–strain curves depicted in Figure S1 and the corresponding mechanical parameters summarized in **Table 2**. The values obtained for the electrospun PVA mat fall within the range reported in the scientific literature [39,40]. The incorporation of MSN into PVA mats significantly improves the stiffness and ductility of PVA electrospun mats. This enhancement is evidenced by the observed increases in Young’s modulus, tensile strength, and elongation at break, indicating a simultaneous improvement in rigidity, load-bearing capacity, and deformability. Such mechanical reinforcement is typically attributed to strong interfacial interactions between MSN and PVA chains, which facilitate efficient stress transfer and restrict polymer mobility. Additionally, the homogeneous dispersion of MSN within the fibrous network contributes to structural integrity and energy dissipation under tensile loading, resulting in a more robust and resilient material. Similar improvements in the stiffness and strength of electrospun PVA mats upon incorporation of reinforcing agents have been previously reported. For example, the addition of MXene flakes [39], copper [41], and silver nanoparticles [28] significantly enhanced both Young’s modulus and tensile strength of PVA. However, these reinforcements also led to a notable reduction in ductility, attributed to the rigid nature of the additives and their influence on polymer chain mobility [28,39,41]. Comparable behavior has been observed in systems incorporating MSN into PLGA and PLGA/gelatin scaffolds, where the enhancement in mechanical properties is attributed to the limited molecular mobility of PLGA in the presence of MSNPs dispersed throughout the nanofiber matrix [42].

In this work, the incorporation of MSNs after crosslinking leads to an increase in material ductility, probably due to the post-crosslinking addition of MSN, which may reduce excessive network rigidity by introducing nanoscale heterogeneities that allow localized deformation, and MSN can act as stress-dissipating domains, mitigating crack propagation and enabling greater elongation before failure. For 3D systems, the mechanical parameters of 3D PVA mats could not be evaluated due to insufficient structural integrity and a lack of cohesive mechanical response under tensile loading. The presence of nanoparticles in the 3D MSN-loaded PVA mats enabled the formation of more robust materials. A reduction of approximately 80% in Young’s modulus, tensile strength, and elongation at break was observed in 3D MSN-loaded PVA mats as compared with 2D MSN-loaded PVA mats, which might be attributed to the highly porous, loosely arranged structures and more MSN content, as observed by SEM-EDS analysis. As expected, the expanded architecture and the presence of voids within the scaffold structure reduce the material’s ability to withstand mechanical stress [21]. Although the expanded materials exhibit lower mechanical properties, they enable higher particle loading and improved antimicrobial activity. Moreover, incorporating a suitable support can help compensate for their limited mechanical integrity and enhance their practical applicability.

**Figure 8:**
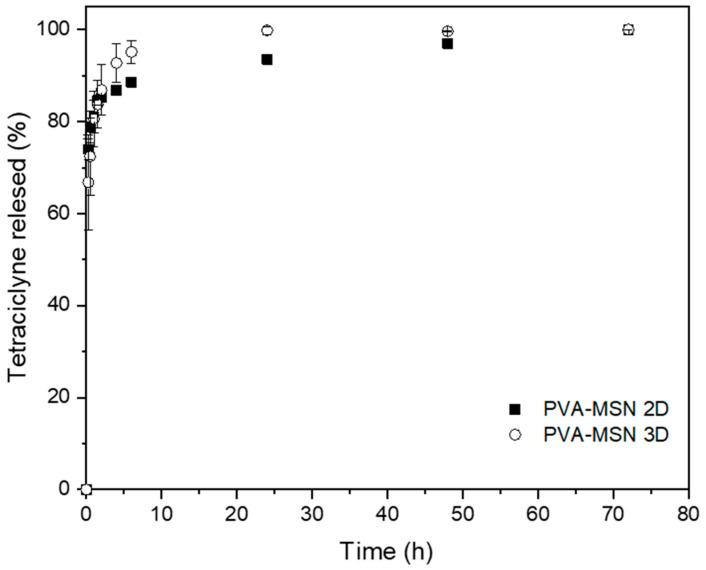
In vitro tetracycline release from 2D and 3D PVA-MSN electrospun mats.

The release kinetics of tetracycline from the 2D and 3D electrospun PVA mats were evaluated (**Figure 8**). The 3D scaffolds exhibited a faster drug release rate compared with their 2D counterparts, a behavior that can be attributed to their higher porosity, larger pore sizes, and enhanced interconnectivity resulting from the gas-foaming process. These morphological features not only facilitated the infiltration of tetracycline-loaded MSN, but also promoted drug diffusion through the fibrous network. The 3D mats exhibited a release profile similar to that of MSN alone, with nearly complete drug release achieved within 24 h, reflecting a minimal diffusional barrier imposed by the expanded matrix. In contrast, the 2D mats exhibited a more prolonged release profile, which is consistent with the denser fiber packing and reduced pore connectivity that generate a polymeric barrier effect, thereby delaying tetracycline diffusion.

**Figure 9:**
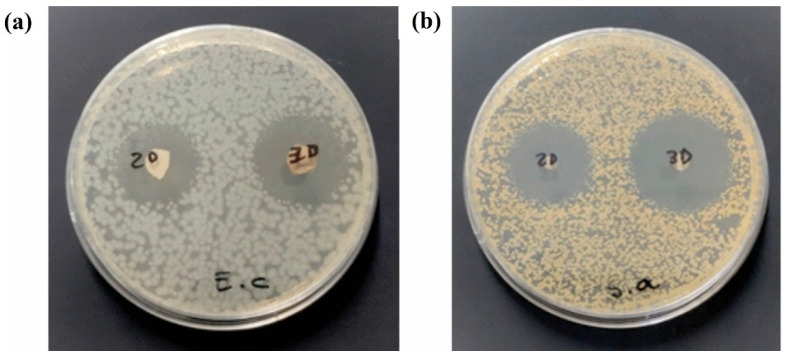
Inhibition zones produced by the 2D (left) and 3D (right) electrospun mats against *Escherichia coli* (**a**) and *Staphylococcus aureus* (**b**). Sample size: 5 mm in diameter.

The antimicrobial properties of the MSN-loaded PVA mats were investigated. Both PVA electrospun mats (2D and 3D) exhibited inhibition halos against the strains *Escherichia coli* ATCC 8739 and *Staphylococcus aureus* ATCC 29213, respectively. **Figure 9a** shows the representative inhibition halos observed in the Petri dishes inoculated with *E. coli*, where the film on the left corresponds to the 2D mat and the one on the right corresponds to the 3D mat. Similarly, **Figure 9b** shows the inhibition halos observed against *S. aureus*, with the 2D electrospun mat placed on the left and the 3D mat on the right. **Table 2** presents the diameters of the inhibition halos along with their corresponding averages for each strain tested. As can be observed, the inhibition halos were enhanced when the 3D electrospun mat was tested, in particular against *S. aureus.* The expanded 3D mats exhibit greater porosity and interconnectivity, enabling higher drug loading and promoting the diffusion of tetracycline into the medium.

Given that inhibition zones were observed in all cases, the assay was continued by collecting samples from the inhibition halos and re-culturing them on tryptic soy agar plates, followed by incubation at 37 °C for 24 h. After the incubation period, no bacterial growth was observed for either *E. coli* or *S. aureus* from the inhibition zones generated by the 2D and 3D scaffolds. These results confirm that both the 2D and 3D membranes exhibit bactericidal activity against the strains *E. coli* and *S. aureus*. Similarly, recent studies using tetracycline-loaded MCM-41 MSN demonstrated their superior inhibition performance against *E. coli* when compared with free tetracycline in culture over a 24 h period [43].

The MIC and MBC assays provided clear evidence of the antimicrobial efficacy of tetracycline released from the electrospun mats. Both 2D and 3D scaffolds showed strong bactericidal activity against *E. coli* and *S. aureus*. For *E. coli*, the MIC and MBC values of tetracycline were 62.5 and 125.0 µg/mL, respectively. For *S. aureus*, the MIC and MBC values of tetracycline were 15.6 and 31.3 µg/mL, respectively. However, the expanded 3D mats demonstrated superior performance, requiring lower material to inhibit and kill bacteria. The MIC and MBC values for *E. coli* based on the mass of the 3D mats were 3.21 and 6.41 mg/mL, respectively, compared with 7.35 and 14.71 mg/mL for the 2D mats. A similar trend was observed against *S. aureus*, with lower MIC and MBC values for the 3D mats (0.80 and 1.61 mg/mL) relative to the 2D mats (1.84 and 3.68 mg/mL). The MIC and MBC values based on the mat’s area are presented in **Table 3**. These results correlate with the higher porosity, increased nanoparticle loading, and faster drug release profile of the 3D scaffolds, which together enhance their antimicrobial activity. Overall, the findings confirm that the gas-expanded nanofibrous mats offer a more effective antibacterial platform than their 2D counterparts, reinforcing the advantages of structural expansion for wound-healing applications.

The conversion of two-dimensional electrospun mats into three-dimensional, gas-expanded scaffolds provides additional advantages, in line with recent reports on gas-foamed fibrous systems. First, the gas-foaming process increases mat thickness, creating an expanded structure. These structural changes allow more mesoporous silica nanoparticles to infiltrate the scaffold, as shown by the higher drug loading in 3D mats (≈1.95 wt% vs. 0.85 wt% in 2D mats) and the stronger Si signal detected by EDS. Second, the expanded pore network facilitates mass transport, enabling a faster tetracycline release from the embedded MSN into the surrounding medium. The higher drug loading and a faster drug release explain the larger inhibition halos observed for the 3D mats when compared with the 2D mats. Taken together, these results demonstrate a direct link between scaffold porosity, drug loading, and drug diffusion, which is consistent with previous studies showing that gas-expanded electrospun mats improve nanoparticle incorporation, fluid absorption, and molecular transport compared with dense 2D mats [21,44,45]. The cytocompatibility of the PVA electrospun matrices employed in this study has already been demonstrated in our recent work, where expanded PVA nanofibers obtained via gas foaming were shown to support fibroblast infiltration and viability [33]. Furthermore, mesoporous silica nanoparticles have been extensively reported in the literature as biocompatible and non-cytotoxic, supporting their use in biomedical applications [29,30].

## 4. Conclusions

In this work, three-dimensional PVA electrospun nanofiber mats incorporating tetracycline-loaded MSN were successfully obtained. The approach combines the obtention of reproducible PVA electrospun mats, the subsequent gas-foaming procedure for their expansion, and the incorporation of antibiotic-loaded MSN. The composite nanomaterial exhibited interesting features, including fibrous morphology, nanometer scale, expanded structure, and thermal stability. FTIR and TGA analyses demonstrated the successful incorporation of the cargo into the MSN. In addition, SEM observations confirmed the infiltration of nanoparticles into the porous mat, showing higher particle loading in the expanded samples, which also influenced the mechanical properties of the material. The antimicrobial activity of the 3D composite material was assessed against both Gram-positive and Gram-negative pathogenic bacteria. The microbiological results confirm that the 3D mats exhibit a better inhibition performance when compared with similar materials in the 2D form. This fact can be explained by the increase in the porosity and interconnectivity produced during the gas-foaming procedure that facilitates the particle infiltration and drug diffusion. The functional materials developed herein have potential application as antimicrobial scaffolds for wound healing. Future research will focus on extending this proof-of-concept study through comprehensive assessments of cytocompatibility and in vivo performance to further support the clinical translation of the developed scaffolds.

## Figures and Tables

**Table 1:** Textural properties of synthesized MSN.

Property	Unit	Value
BET-specific surface area	m^2^ g^−1^	1143
Micropore-specific surface area	m^2^ g^−1 a^	0
Mesopore-specific surface area	m^2^ g^−1^	1143
Total pore volume	cm^3^ g^−1^	0.699
Micropore volume	cm^3^ g^−1^	0
Mesopore volume	cm^3^ g^−1 a^	0.699
Average mesopore diameter	nm	2.87

^a^ Micropore surface area = BET specific surface area–mesopore surface area. Mesopore volume = total pore volume–micropore volume.

**Table 2:** Mechanical properties of 2D and 3D electrospun PVA mats incorporating MSN.

Material	E (MPa)	Elongation at Break (%)	Tensile Strength (MPa)
PVA-2D	93.11 ± 5.34 ^a^	12.70 ± 3.40 ^a^	6.10 ± 1.80 ^a^
PVA-MSN 2D	107.74 ± 5.54 ^b^	20.40 ± 3.0 ^b^	9.03 ± 1.12 ^b^
PVA-MSN 3D	34.11 ± 5.66 ^c^	3.95 ± 0.33 ^c^	1.98 ± 0.60 ^c^

* Different letters in the same column indicate significant differences (*p* < 0.05; Tukey test).

**Table 3:** Inhibition halos, MIC, and MBC values of 2D and 3D electrospun mats against *E. coli* and *S. aureus*.

Material	*E. coli*			*S. aureus*		
	Inhibition Halos (mm)	MIC (cm^2^/mL)	MBC (cm^2^/mL)	Inhibition Halos (mm)	MIC (cm^2^/mL)	MBC (cm^2^/mL)
**2D mat**	26. 7 ± 2.5	2.89	5.77	25.0 ± 1.0	0.72	1.45
**3D mat**	27. 7 ± 2.1	1.26	2.52	29. 3 ± 2.2	0.31	0.63

MIC and MBC determinations were consistent among samples. Sample size: 5 mm in diameter.

## Data Availability

The datasets generated and/or analyzed during the current study are available from the corresponding author upon reasonable request.

## Supplementary Materials

The following supporting information can be downloaded at: https://www.mdpi.com/article/10.3390/pharmaceutics17101335/s1, Figure S1: Stress-strain curves for PVA and PVA-MSN systems in 2D and 3D.
