# Supplementary figures and images for: Expanded Nanofibrous Polymeric Mats Incorporating Tetracycline-Loaded Silica Mesoporous Nanoparticles for Antimicrobial Applications
Source: Pharmaceutics. 2025 Oct 15;17(10):1335. doi: 10.3390/pharmaceutics17101335 (PMC12567016; doi:10.3390/pharmaceutics17101335)

## SUPPLEMENTARY INFORMATION

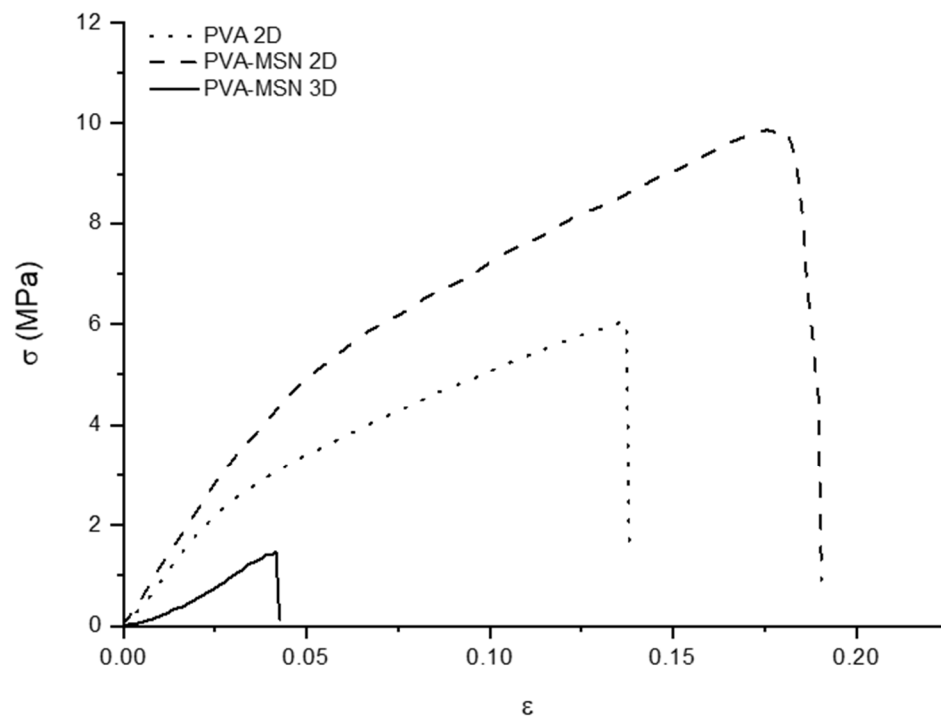

**Figure S1.** Stress-strain curves for PVA and PVA-MSN systems in 2D and 3D.

Supplement: Supplementary file 1 [file pharmaceutics-17-01335-s001.zip › pharmaceutics-3874632-supplementary.pdf]
